# Functionalized and Theranostic Lipidic and Tocosomal Drug Delivery Systems: Potentials and Limitations in Cancer Photodynamic Therapy

**DOI:** 10.34172/apb.2024.038

**Published:** 2024-03-11

**Authors:** Fahime Nasr Esfahani, Sahand Karimi, Zahra Jalilian, Mehran Alavi, Bushra Aziz, Enam Alhagh Charkhat Gorgich, M. R. Mozafari, Elham Taghavi, Sargol Aminnezhad, Sara Ataei

**Affiliations:** ^1^Australasian Nanoscience and Nanotechnology Initiative (ANNI), Monash University LPO, Clayton, VIC 3168, Australia.; ^2^Department of Biological Science, Faculty of Science, University of Kurdistan, Sanandaj, Kurdistan 6617715175, Iran.; ^3^Department of Physics, Women University of Azad Jammu & Kashmir, Bagh 12500, Azad Kashmir, Pakistan.; ^4^Department of Anatomical Sciences, School of Medicine, Iran University of Medical Sciences, Tehran 1449614535, Iran.; ^5^Faculty of Fisheries and Food Science, Universiti Malaysia Terengganu (UMT), 21030 Kuala Nerus, Terengganu, Malaysia.; ^6^Department of Microbiology, Faculty of Biological Sciences, Alzahra University, Tehran, Iran.; ^7^Department of Clinical Pharmacy (Pharmacotherapy), Tehran University of Medical Sciences, Tehran, Iran.

**Keywords:** Photodynamic therapy (PDT), Photosensitizer (PS), Medicinal plants, Drug targeting, Vesicular carriers, Cell-based drug delivery systems

## Abstract

Photodynamic therapy (PDT) is a multidisciplinary area, which involves photophysics and photochemical sciences and plays an important role in cancer diagnosis and treatment. PDT involves a photo-activable drug called photosensitizer (PS), a specific wavelength of light and cellular compounds to produce toxic oxygen species in a much-localized way to destroy malignant tumors. Despite the various benefits of PDT, some PS-related limitations hinder its use as an ideal treatment option for cancer. To address these limitations (e.g., poor bioavailability, weak permeability, hydrophobicity, and aggregation), lipid-based and vesicular drug delivery systems have been employed. These carrier systems possess the ability to enhance the bioavailability, permeability, and solubility of the drug. Furthermore, they tend to load hydrophobic and lipophilic compounds and can be employed for an efficient and targeted drug delivery. The purpose of this review is to highlight the precise idea of PDT, the limitations of PDT related to PS, and the application of lipidic and tocosomal carriers in PDT for the treatment of various types of cancers. Liposomes, nanoliposomes, solid lipid nanoparticles, vesicular phospholipid gels, exosomes, transferosomes, and tocosomes are presented as commonly–employed vesicular drug carriers. Moreover, the amalgamation of cell-based drug delivery systems (CBDDS) with PDT holds considerable potential as an encouraging avenue in cancer treatment, especially in the context of immunotherapy.

## Introduction

 Cancer is a leading cause of death worldwide with a significant impact on human health and well-being.^1,2^ Conventional cancer treatment strategies including surgery, chemotherapy, and radiotherapy have been used to combat this deadly disease.^3^ However, these strategies still have adverse side effects, poor tumor targeting, and low survival rate.^4,5^ These drawbacks make them far away from being ideal treatment options.^6^ Photodynamic therapy (PDT) was introduced in the 19th century as an alternative cancer treatment and has shown promise due to its non-invasive nature and possibility of localized, site-specific therapy.^7^ While initially identified for its bactericidal properties, it was later explored as a therapeutic intervention for cancer.^8^ PDT procedures involve the use of a specific photo-activated drug, which, upon exposure to a particular wavelength of light in the presence of cellular molecular oxygen, becomes toxic to cancer cells. Referred to as a photosensitizer (PS), this photo-activated drug remains non-toxic to cells in the absence of illumination. This particular attribute stands out as one of the most appealing features of PDT. The activated PS generates reactive oxygen species (ROS), which can eliminate malignants cells. The selectivity for tumor cells relies on the higher retention of generally lipophilic PSs in malignant cells compared to healthy cells. Additionally, malignant cells exhibit greater susceptibility to oxidative stress, leading to higher mortality when exposed to such stress compared to healthy cells. The prolonged accumulation of PSs in the neoplasm region is attributed to inadequate lymphatic drainage and heightened permeability of blood vessels.^9^

 Although PDT has considerable benefits as compared to other conventional cancer treatment options, some disadvantages are associated with the PS. The most common disadvantage associated with PS is its poor bioavailability.^10^ In this perspective, nanotechnology plays a vital role in cancer treatment through the development of nano drug delivery carriers which can increase the stability, bioavailability, and efficacy of the encapsulated drug. Among the available drug delivery systems lipid-based carriers have shown potential benefits, particularly concerning biocompatibility, prolonged blood circulation, time and high drug permeability through cellular membranes.^11^ These carriers can load not only hydrophobic and hydrophilic drugs but also amphiphilic compounds. This feature makes them ideal for various therapeutic and industrial applications. The bioavailability, specificity, and solubility of PS in PDT could be improved by employing lipidic drug delivery systems.^12^ The purpose of this review article is to highlight the role and benefits of using lipid-based carriers for the encapsulation of PS compounds to be used in the PDT of various cancers. The most recently developed vesicular carrier system known as tocosome is also described within this context.^13^

## PDT: an alternative treatment option for cancer

 PDT is considered an alternative treatment option for many types of cancer. It employs three patient-friendly, non-invasive, components to combat cancer. These components are: (*i*) a light-activated drug called PS, (*ii*) specific wavelength of light, and (*iii*) molecular oxygen.^14^ PS accumulates in the tumor site either by passive or active transport. It is activated by a specific wavelength of light to kill the cancer cells via photophysical and photochemical reactions. Activated PS jumps from the ground state to the excited state (short lifetime and higher energy), from where jumps into the triplet excited state (lower energy and long lifetime), via intersystem crossing, and subsequently acts as a catalyst in photochemical reactions. At the triplet excited state, two photochemical reactions (i.e., type-I, and type-II PDT) occur. Type-I reaction involves the transfer of PS energy to a nearby bio–substrate to produce hydrogen peroxides, hydroxyl radicals, superoxide anion, and ROS. In type-II PDT, the triplet excited state PS energy is transferred into triplet excited state of molecular oxygen, which further causes the formation of singlet oxygen (highly toxic agent). In both of the PDT types, cell death is caused by necrosis, apoptosis, autophagy, and activation of the immune system.^14,15^ PDT depends upon the type of PS, PS uptake, light exposure, and oxygen concentration. The most important feature of PS, which plays a significant role in cancer cell death, is its subcellular uptake by different organelles. PDT has various advantages over other conventional treatments, e.g., low toxicity, selectivity, localization, and minimal tissue penetration.^16^ In the late 1970s, the first clinically applied PS for PDT was hematoporphyrin derivative and by now several PSs have been approved. These include talaporfin sodium, verteporfin, Foscan, and 5-aminolevulinic acid (ALA).^14,17^

**Figure 1:**
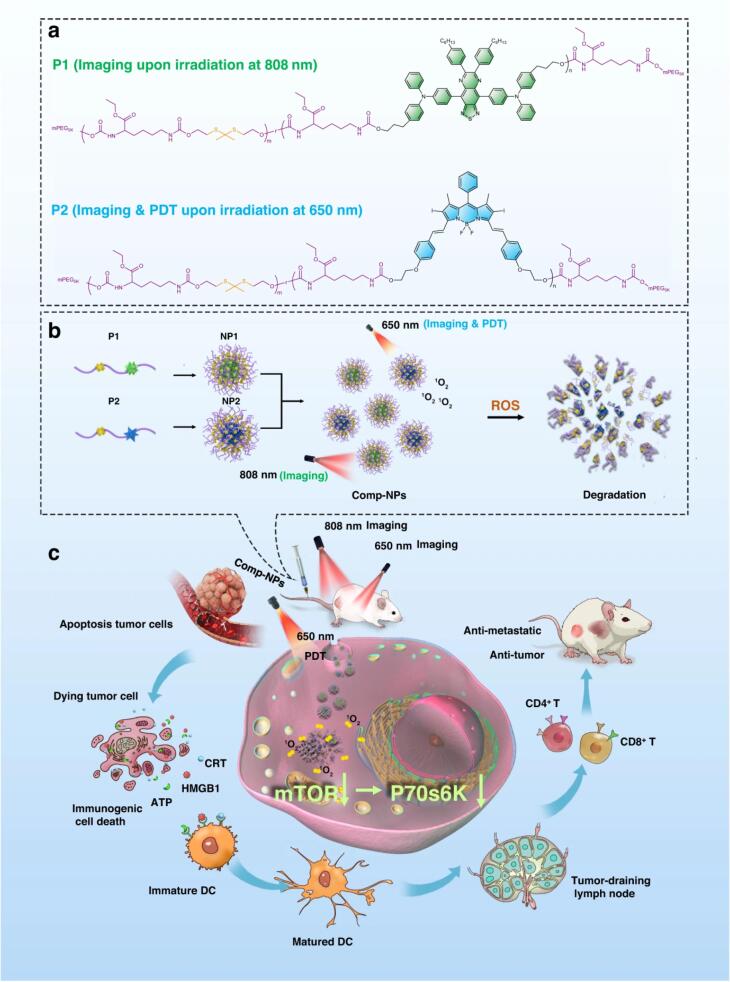
The figure illustrates the structures and mechanism of action of Comp-NPs for tumor diagnosis through imaging and treatment via multimodal photodynamic therapy (PDT) and immunotherapy. (a) Chemical structures depict a polymer with a chromophore (P1) for imaging at irradiation of 808 nm or a photosensitizer (P2) for PDT at 650 nm irradiation. (b) Self-assembly of these polymers into nanoparticles (NP1 and NP2) leads to the formation of the theranostic nanoparticle formulation Comp-NPs. (c) The biological mechanism demonstrates Comp-NPs' action through combined PDT and immunotherapy (Adopted from Zhou et al^18^ under a Creative Commons Attribution 4.0 International License)

 Zhou and colleagues introduce a theranostic nanomaterial for efficient tumor diagnosis and treatment. These nanoparticles remain stable in physiological conditions but degrade under ROS, containing a photosensitizer for breast cancer therapy in cells, tumor spheroids, and mice. Using an 808 nm chromophore enables tumor detection. Mechanistically, the nanomaterial induces immunogenic cell death (ICD) in cancer cells and animal models, reducing primary tumor volume and eradicating metastases. It inhibits growth in multi-drug resistant hepatocellular carcinoma and interacts with the mTOR (mechanistic target of rapamycin) signaling pathway pivotal in tumor evolution. Targeting mTOR holds promise for anticancer strategies. Additionally, the study elucidated nanocomposite impact on signaling pathways using GOcircos and KEGG (Kyoto Encyclopedia of Genes and Genomes) analyses, highlighting disruptions in key pathways crucial for tumorigenesis and metastasis. Nanocomposite, along with light exposure, significantly perturbed pathways essential for cancer development and spread, including p53-mediated signal transduction, cell cycle regulation, and cell adhesion. Moreover, they disrupted mTOR and eIF4/p70S6K signaling, pivotal for tumorigenesis and metastasis (Figure 1).^18^

## Limitations of PDT

 The primary clinical limitations of PDT for cancer therapy are outlined in Table 1.^16^ PDT offers notable advantages over traditional cancer treatment methods, including the absence of significant side effects, minimal invasiveness, and high specificity for tumor sites. However, a drawback of PDT is its limitation to superficial oncologic lesions with a tumor thickness of less than 2 to 3 mm.^19^ This limitation arises from the fact that light within the visible wavelength range possesses restricted tissue penetration capabilities.^20^ Furthermore, some PS-related drawbacks are present, which limit the efficacy of the PDT strategy. The hydrophobicity is the major PS-related drawback, and in tumor sites, these hydrophobic compounds leak out of the circulatory system. In some cases, these compounds undergo self-aggregation in biological media, which results in poor bioavailability, impaired solubility, and off-target activation. This poor pharmacokinetic behavior affects the photophysical and photochemical process of PDT.^21^ To optimize efficiency, site-specific targeting and in general the pharmacokinetic behavior of PS molecules, lipid-based drug delivery systems can be employed. These carrier systems are used to encapsulate bioactive agents and can also transport PS molecules to their target sites more efficiently. In particular, drug delivery systems effectively improve the transport, stability, and bioavailability of the encapsulated agents.^22^

**Table 1:** The main clinical limitations of PDT application in cancer therapy^16^

**Limiting factor **	**Description **	**Overcoming limitations **
Light	The penetration of light is affected by the optical properties of the wavelength and the tissue (Figure 2A). Generally, the applied light in PDT can penetrate tissue by a few millimeters depth.	The application of lasers, provide a monochromatic light with appropriate high-power results.
Oxygen	The cancer tissues have limited oxygen caused by insufficient vasculature and rapid growth. This hypoxic condition of tumor tissue reduces the efficiency of PDT.	Combination of PDT with hyperbaric oxygen (HBO_2_) therapy.
Physicochemical properties of PS	Low hydrophilicity and aggregation in aqueous medium.	1) Insoluble PSs can be loaded on nanoparticles or encapsulated in liposomes and emulsions. 2) Binding hydrophilic substituents to PSs. 3) Synthesis of nonionic hydrophilic PSs by modification with functional groups such as polyhydroxylate and carbohydrate.

## PDT and effects on immune system

**Figure 2:**
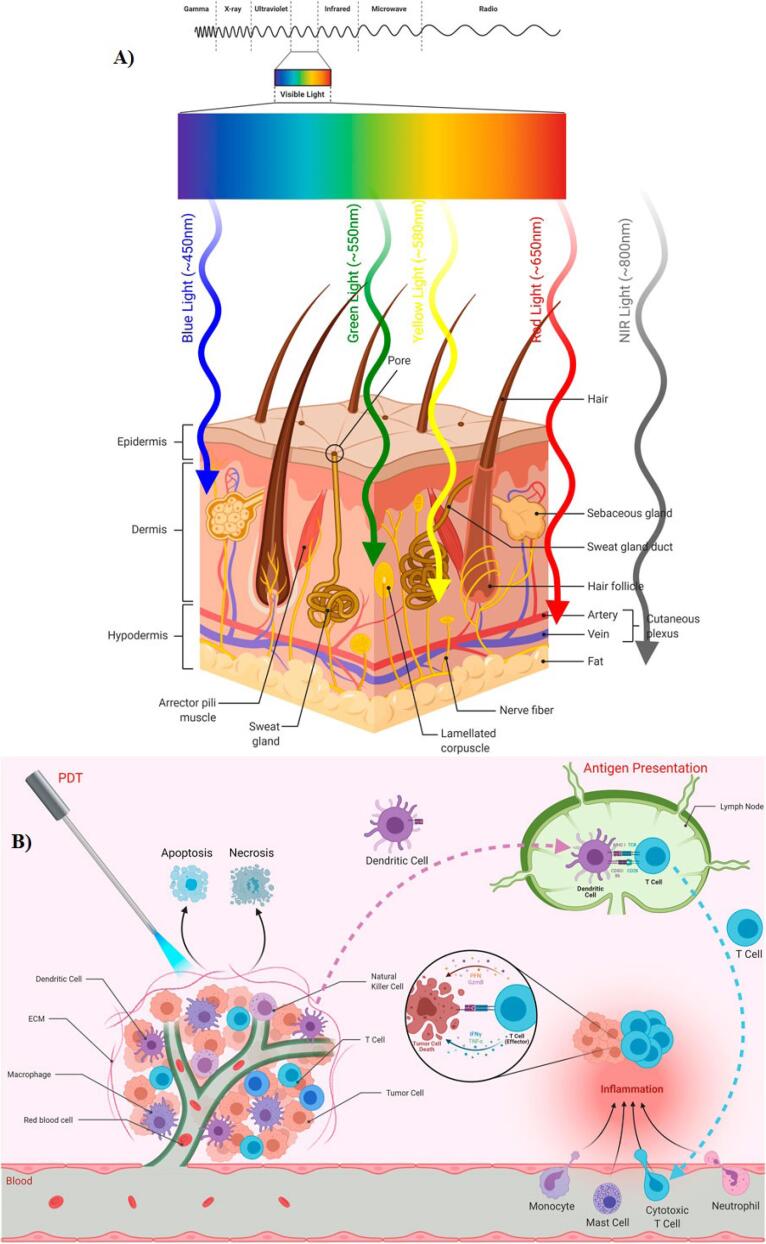
(A) Depth of light penetration into skin based on its wavelengths, (B) PDT influences immune responses, fostering anti-tumor immunity while also promoting inflammation at the targeted tissue. Adopted from Gunaydin et al^16^ under the terms of the Creative Commons Attribution License (CC BY)

 PDT sparks robust immune responses, fueling anti-tumor activity and inflammation. By inducing tumor cell death, it activates immune reactions against tumor antigens, recruiting effector cells to the tumor site. Immune cells like dendritic cells present these antigens to T cells, recruiting effector cells like T cells, monocytes, mast cells, and neutrophils to the tumor site. PDT’s ability to trigger acute inflammation is crucial for anti-tumor immunity, evidenced by increased cytokines and leukocyte infiltration. CD8 + T cells and NK cells are key in preventing tumor regrowth post-PDT. However, immune suppression may occur, influenced by light irradiation factors (Figure 2B).^16^

 During a Phase-I PDT trial, patients experienced systemic capillary leak syndrome. They underwent either pleurectomy or extrapleural pneumonectomy followed by intraoperative PDT using Foscan and red light. Analysis of serum samples after treatment revealed heightened levels of IL-1beta, IL-6, IL-8, and IL-10 following both surgery and PDT, indicating systemic inflammation. However, levels of IFN-gamma, TNF-alpha, and IL-12 remained unaltered. Notably, IL-1beta notably increased post-surgery, while IL-6 surged post-PDT, suggesting a cytokine-mediated response. These findings underscore the necessity for further exploration into the underlying mechanisms.^23^

## The role of nanotechnology in enhancing PDT efficacy

 Outcomes of cancer imaging and therapy have been affected significantly by the discovery of novel organic and synthetic nanomaterials.^15,24^ Advancements in nanotechnology have significantly improved the overall efficacy of PDT. PS-related limitations are addressed with the help of nano-based pharmaceutical formulations including, lipid-based (e.g., liposomes, nanoliposomes, exosomes), surfactant-based (niosomes), polymer-based (polymeric nanoparticles, micelles, dendrimers, nanogels), and inorganic (silver, gold, iron, ZnO, silica, quantum dots) nanosystem.^24-26^ The advantages of nano-pharmaceutical formulations vary depending on their type and preparation protocol. However, they also possess the following benefits:

- Surface modification for targeted therapy.^27,28^
- Enhance the biodistribution of the encapsulated agents;
- Ability to take benefit of enhanced permeability and retention effect for PS-loaded system in tumors cells.^29,30^
- Decrease nonspecific targeting and decrease or eliminate side effects.^31^
- Multi-functional theranostics can be achieved by co-loading PS, other drugs, and imaging contrasts; and:
- Ability to load different therapeutic drugs in combinational strategies.^32,33^

The advantages and limitations of conventional PDT protocols and nanotechnology-based PDT are listed in Table 2.

**Table 2:** Comparison between conventional PDT procedures and nanotechnology-based PDT with respect to their limitations/benefits^34^

**Conventional PDT**	**Nanotechnology-based PDT**
Drug resistance	Combined therapies
Harmful radiation	Enhanced imaging
Impaired combination therapies	Fast diagnosis
Incoherent pharmacokinetic & pharmacodynamic profiles	Increased therapeutic efficiency
Late-stage diagnosis	Limited radiation exposure
Systemic Side effects	Lowered toxicity
	Multi-functionality
	Theragnostics

 As mentioned in the above section, with limited tissue penetrability of light, the therapy can induce severe pain, hypoxia, and wide-ranging tumor resistance. The main obstacles to the clinical application of PDT are the brief half-life of photosensitizers in plasma and the ineffective induction of tumor cell death. In this way, nanoformulation based on biocompatible, bioavailable, and biodegradable organic and inorganic nanomaterials can be used as nanocarriers of photosynthesized. Nanocarriers can address the limitations of traditional photosensitizers by responding to a broad spectrum of light sources, making them suitable for treating deep-seated tumors. Moreover, modulation of cell death pathways, reduced drug resistance, and pain in PDT can result from the combination therapy of anticancer and photosensitizer agents.^35^ In conventional PDT, the emergence of drug resistance and decreased therapeutic effects can be caused by locally-induced hypoxia in the tumor microenvironment. In addition to photosensitizer delivery nanosystems, several strategies involving nano-based photoactive drugs, ROS-tunable photosensitizers, and organelle targeting have been presented for the reduction of locally-induced hypoxia.^36^ For example, loading hypoxia-activable prodrug tirapazamine on polyvinyl pyrrolidone dispersed metal-organic framework aggravated tumor hypoxia and synergized chemotherapy effect of the tirapazamine.^37^ A biodegradable and biocompatible bismuthene/bismuth oxide (Bi/BiOx) nanostructure significantly produced cytotoxic H_2 _and •OH under hypoxia under irradiation at 660 nm. Moreover, there was improved tumor tissue penetration and higher cellular uptake for this formulation compared to conventional PDT.^38^

## Lipid-based drug delivery carriers

**Figure 3:**
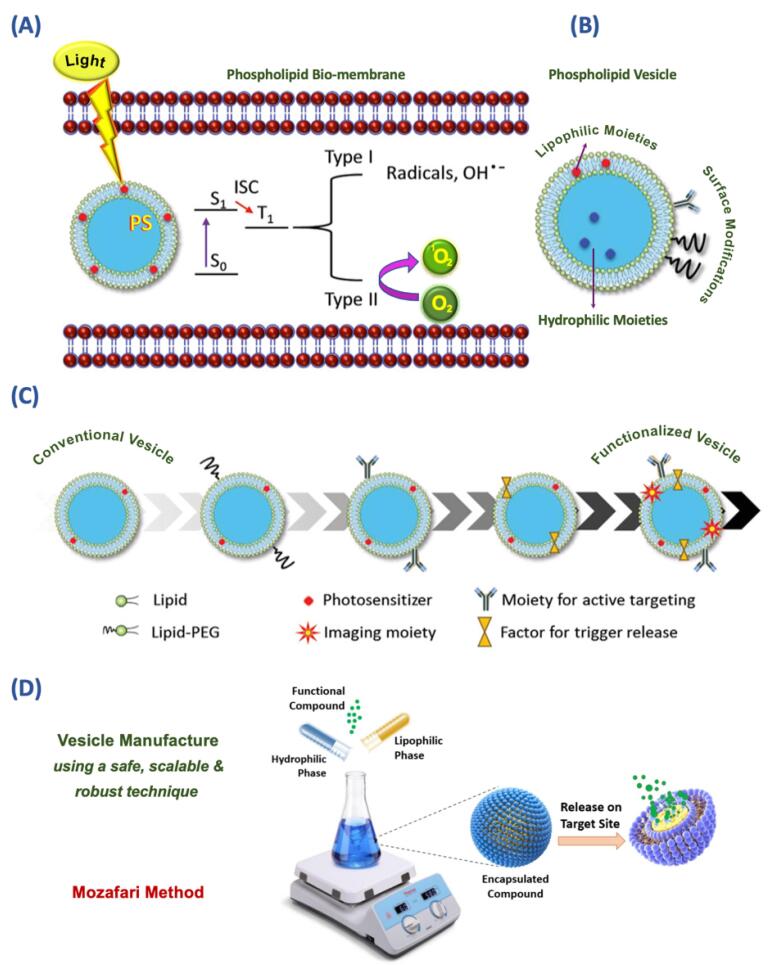
(A) Type I and II processes of cytotoxicity induced by light-activated photosensitizer (PS). Encapsulated PS is activated from the ground state (S_0_) to the excited state (S_1_) upon light irradiation causing cell death by inducing radicals and ROS. (B) Structure of a drug delivery vesicle that consists of the phospholipid bilayer, aqueous core, and lipophilic molecules embedded in the lipid bilayer, hydrophilic molecules encapsulated inside the aqueous core, recognition moieties, and polyethylene glycol (PEG) linker on the vesicle surface. (C) Development of functionalized/theranostic vesicles from a simple/conventional vesicle. (D) Mozafari method as a simple and green technology for the preparation of lipid-based vesicles and other encapsulation systems (Partially taken and highly modified with permission from ^9,22^)

 Lipidic carrier systems are advanced drug delivery protocols featuring biodegradability, biocompatibility, nontoxicity, and targetability. Of particular interest are liposomes, which are vesicles composed of one or more lipid/phospholipid bilayer(s) enclosing central aqueous compartments and have been widely used for biomedical applications as well as in food, nutraceutical, and cosmetic industries.^39-41^ These lipid vesicles can selectively deliver their encapsulated substances into the target cells/tissues through passive or active transport, therefore reducing the adverse side effects, and enhancing the therapeutic outcomes.^42^ Based on vesicle structure, liposomes are generally classified into the following the categorization comprises 1) unilamellar vesicles (ULVs) and 2) multilamellar vesicles (MLVs). ULVs are further classified into small unilamellar vesicles (SUVs), large unilamellar vesicles (LUVs), and giant unilamellar vesicles (GUVs).^43^ However, other types of lipid vesicles such as double-bilayer vesicles and multivesicular vesicles have also been synthesized. Liposomes have the potential to simultaneously entrap the hydrophilic, hydrophobic, and amphiphilic molecules, which gives them an edge over alternative drug delivery techniques.^44^ The liposomal structure’s unique molecular arrangement allows hydrophilic compounds to be encapsulated in the vesicle’s core while incorporating hydrophobic molecules into the lipid bilayer (Figures 3A and B).^42^ Based on their unique characteristics, several marketed approved nanomedicines are based on liposomes or lipid nanoparticles. Table 2 lists some of the already approved pharmaceutical products on the market that are based on lipidic carrier systems.^45^ This shows that lipidic carriers are not only research and development tools confined to the laboratories but also an essential component of the high-tech pharmaceutical products on the market. Consequently, they have great potential to be approved for the encapsulation of PS molecules in clinical PDT procedures.

 As shown in Table 3, VISUDYNE^®^ received FDA approval in 2000 as a liposomal formulation containing the benzoporphyrin analog monoacid ring A, serving as a PS.^46^ Prescribed for the treatment of choroidal neovascularization resulting from age-related wet macular degeneration, VISUDYNE^®^ is administered intravenously. This condition, characterized by the proliferation of undesired blood vessels in the back of the eye, is a leading cause of adult blindness. Following administration, a red laser is directed through the eye pupil after a 10-minute interval. The PS in VISUDYNE^®^ absorbs light, entering an excited state, transferring energy to ambient oxygen, and producing singlet oxygen. This ROS then damages the newly formed leaky blood vessels, halting and reversing progressive vision loss. Beyond its use for macular degeneration, a combination of VISUDYNE^®^ PDT and immunosuppression is recommended for treating sub-foveal choroidal neovascularization resulting from inflammatory conditions. Typically, side effects of VISUDYNE^®^ treatment are moderate, encompassing slight vision changes, light flashes, headaches, and mild eye dryness, redness, or swelling.^47^

**Table 3:** Marketed liposome-based carrier technology approved for human use with drug loading^48^

**Brand name**	**Loaded drug **	**Therapeutic applications**
Doxil^®^	Doxorubicin	Chemotherapy drug (ovarian cancer, multiple myeloma, and Kaposi's sarcoma)
AmBisome^®^	Amphotericin B	Antifungal medication
VISUDYNE^®^	Verteporfin	Treatment of certain eye conditions (age-related macular degeneration)
DepoDur^®^	Morphine sulfate	Extended pain relief following surgery
Marqibo^®^	Vincristine sulfate	Chemotherapy drug (Philadelphia chromosome-negative acute lymphoblastic leukemia)
VYXEOS^®^	Daunorubicin and cytarabine.	Combination of the chemotherapy drugs (certain types of acute myeloid leukemia)

###  Nanoliposomes

 With the rapid advancements in nanoscience and nanotechnology, the term ‘nanoliposome’ has been coined to specifically denote nanoscale lipid-based vesicles and to distinguish them from their micrometric counterparts (i.e., liposomes). It is important to note that ‘liposome’ is a broad term encompassing various classes of lipid vesicles within the size range of hundreds of nanometers to several micrometers.^49^ In general, liposomes and nanoliposomes exhibit similar structural and thermodynamic properties, primarily dictated by their ingredients and suspension media. However, the reduction in particle size results in larger surface-to-volume ratios for nanoliposomes. Consequently, compared to liposomes, nanoliposomes offer increased surface area, potentially enhancing solubility, improving bioavailability, facilitating controlled release, and enabling more precise targeting of encapsulated molecules. The synthesis process for both liposomes and nanoliposomes involves the input of energy into a dispersion of lipid and phospholipid molecules in an aqueous medium.^50^ The formation of both liposomes and nanoliposomes is driven by Van der Waals forces and hydrophilic-hydrophobic interactions between phospholipids and water molecules. Within the structure of lipid vesicles, non-covalent interactions, including electrostatic forces, depletion forces, and steric interactions, play a significant role in the interactions between lipids/phospholipids and the encapsulated material.^51^ Lipid vesicles are dynamic structures prone to aggregation and fusion, leading to an increase in size over time. Consequently, vesicles initially prepared in nanometric size ranges may transform into micrometric vesicles during storage. However, it is essential for nanoliposomes to maintain an acceptable stability profile to preserve their sizes within nanometric scales throughout the shelf-life of the pharmaceutical product. A scientifically robust definition for nanoliposomes could thus be: “bilayer lipid vesicles possessing and sustaining nanometric size ranges during both storage and application.^52^

 Research and development of nanoliposomal PS formulations are ongoing at laboratories in different parts of the world. Liposomes and nanoliposomes used for PDT applications have experienced several stages from conventional/simple vesicles to functionalized vesicles as depicted in Figure 3C. In a recent study, Chen and colleagues synthesized two zinc (II) phthalocyanines, known as potent PS, each monosubstituted with a sulphonate group in the alpha position with either an “O bridge” or an “S bridge.” They then prepared a nanophotosensitizer using the thin-film hydration method to control the aggregation of PS molecules in an aqueous solution and enhance their tumor-targeting capability. The resulting nanoliposomal formulations demonstrated highly efficient generation of superoxide radicals and singlet oxygen under light irradiation, exhibiting a 2.6-fold and 15.4-fold increase compared to unencapsulated PS, respectively. Additionally, these formulations displayed selective accumulation in tumors after intravenous injection, showcasing their potential for enhanced tumor targeting in PDT.^53^ The main drawback of the thin-film hydration method used in the study for the preparation of lipid vesicles is the employment of potentially toxic solvents such as chloroform and methanol.^53^ However, currently there are methods available for the large-scale manufacture of lipidic and vesicular carrier systems, which do not require the utilization of any toxic solvent, detergent, or harsh treatments such as high-pressure homogenization. Examples of these green technologies include a heating method,^54^ and the Mozafari method (Figure 3D).^55,56^

## Role of drug delivery systems in PDT

**Figure 4:**
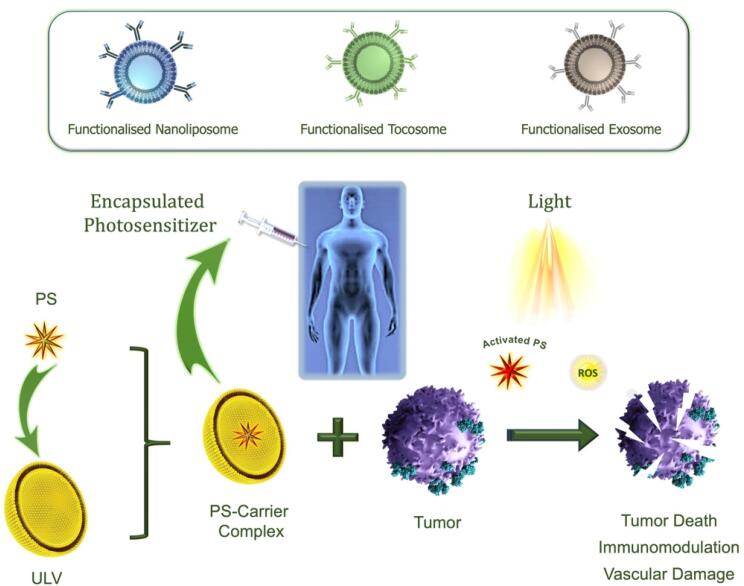
Simplified illustration of the mechanism of action of PDT using encapsulated PS. A ULV can be used to encapsulate PS and target it to a tumor site. Upon exposure to visible light of an appropriate wavelength, a PS molecule transitions from its ground singlet state to an activated state. In the presence of molecular oxygen, the excited PS generates ROS, initiating a cascade of reactions with surrounding organic molecules. This oxidative damage ultimately results in cell death. Antibody-grafted nanoliposomes, tocosomes, or exosomes can be used as functionalized drug carriers for the encapsulation of PS^9^

 In recent years, several carrier systems have been developed to enhance the selectivity and optimize the bio-distribution of various drugs, vaccines, and other bioactive agents. A number of these drug delivery technologies were also used for the encapsulation and controlled release of PS in PDT. Due to the numerous benefits and potential uses of lipidic carriers, they have been used in PDT applications for the encapsulation of a variety of PS molecules.^57^ A simplified mechanism of action of encapsulated PS towards cancer eradication is depicted in Figure 4.

 They were used for a variety of purposes in PDT applications, such as decreasing side effects, enhancing selectivity, enhancing PS phototoxicity, and reducing immunogenicity, which resulted in higher PDT efficiency. A hydrophobic chlorin-like photosensitizer called verteporfin has been demonstrated to be extremely efficient for PDT *in vivo*. In aqueous conditions, verteporfin also has a propensity for self-aggregation, which can significantly reduce the drug’s bioavailability to biological systems. Because it is crucial to administer verteporfin to the body in its monomeric state, it was put into liposomes for intravenous medication delivery.^58^ For the PDT of age-related macular degeneration, VISUDYNE^®^ was the only medication that the FDA has approved. Without endangering the nearby tissues, the VISUDYNE^®^ procedure stops the formation of harmful blood vessels.

 A total of 609 patients diagnosed with age-related macular degeneration were enrolled in both phase I and phase II clinical trials.^59^ A significant challenge in PDT is the hypoxic tumor microenvironment. An innovative approach involves utilizing liposomes loaded with Chlorin e6 photosensitizer, a hypoxia-activated prodrug tirapazamine, and a gene probe. In both in vitro and in vivo studies, the outcomes demonstrated improvement compared to traditional PDT, addressing the issue of the hypoxic tumor microenvironment.^60^ Different liposomal formulations encapsulating temoporfin (second-generation, synthetic, effective PS) with increased phototoxicity against SK-OV-3 cancer cells have been developed. When SK-OV-3 cancer cells were exposed to three liposomal formulations encapsulating temoporfin and 10 J/cm^2^ of LED light, their cell viability was decreased to 20%. All of the developed liposomal formulations also demonstrated hemocompatibility (10% hemolysis) and a coagulation time of less than 40s.^61^

 AlPcS4 is a promising PS that has various benefits, including good quantum yields, significant tissue penetration, appropriate photostability, and minimal photobleaching. Because of its poor release efficiency and strong binding affinity to serum albumin, it has little penetration into cancer cells. Cationic liposomes have been employed in an effort to overcome this drawback. AlPcS4 (aluminum phtalocyanine chloride tetrasulfonic acid) safety and effectiveness can be improved when it encapsulated in a liposomal drug delivery system.^62^ AlPcS4 encapsulating in the liposomal carriers has also been improved the PDT effectiveness and decrease PS affinity for binding to serum proteins.^63^ In comparison to free and non-targeted liposomal AlPcS4, the formulation of AlPcS4-loaded transferrin-conjugated PEG-liposomes exhibited improved tumor-selective accumulation in bladder tumor tissues. Additionally, this same AlPcS4-loaded transferrin-conjugated PEG liposomal system demonstrated successful application in the treatment of cervical cancer.^64^

## Cell-based drug delivery systems (CBDDS) and PDT

 The integration of CBDDS with PDT signifies a state-of-the-art and synergistic approach in the field of medical treatment. This innovative combination harnesses the unique advantages of both technologies to enhance the precision, effectiveness, and versatility of therapeutic interventions. In the pursuit of enhanced cancer immunotherapy, the integration of CBDDS systems and PDT plays a pivotal role in the cancer immunity cycle. CBDDS utilizes living cells as carriers to deliver immunotherapeutic agents with precision, targeting tumor cells and bolstering the immune response. Simultaneously, PDT employs photosensitizing agents and light to selectively destroy cancer cells, triggering immune activation and fostering immunological memory. Together, these integrated approaches optimize antigen presentation, immune activation, and memory development, offering a promising path toward improved cancer treatment by harnessing the immune system’s capabilities to combat cancer effectively. This integrated approach holds promise for a wide range of medical applications, from cancer treatment to addressing challenging anatomical locations and complex diseases. However, it is an evolving field, and thorough research and development efforts are required to fully understand its potential benefits and overcome associated challenges, including safety considerations and regulatory hurdles.^65-67^

**Figure 5:**
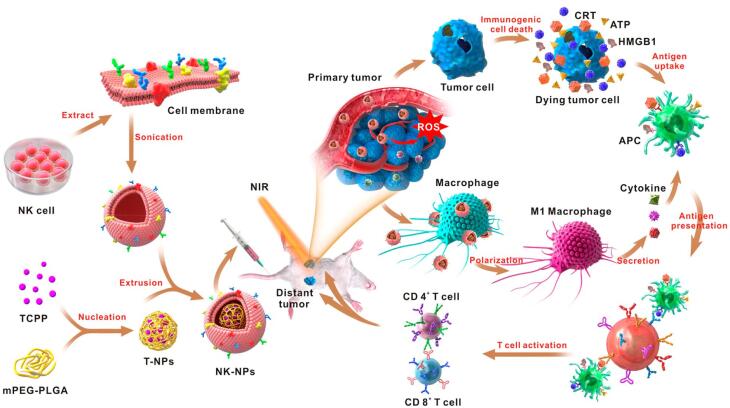
Schematic representation of nanoparticles coated with NK cell membranes, showcasing their role in enhancing cell-membrane immunotherapy through PDT. Reprinted with permission from Deng et al^68^; Copyright (2024) American Chemical Society.

 The research conducted by Deng et al proved the synergistic application of PDT alongside diverse strategies to trigger programmed cancer cell death, encompassing apoptosis and necrosis, in cancer cells.^68^ These approaches include using nanoparticles coated with natural killer (NK) cell membranes to selectively target tumors, induce pro-inflammatory responses, and improve the overall immune response against cancer. In this research, coated polymeric nanoparticles were loaded with the 4,4′,4′′,4′′′-(porphine-5,10,15,20-tetrayl) tetrakis (benzoic acid) (TCPP) as PS with NK cell membranes using extrusion. This NK cell membrane coating endowed the resulting NK-NPs with the ability to trigger a pro-inflammatory response, specifically M1-macrophage polarization, within the tumor. This, in turn, facilitated the development of a cell-membrane-based immunotherapy. NK-NPs were also capable of inducing dying tumor cells to release damage-associated molecular patterns (DAMPs) through PDT-induced ICD. These DAMPs included exposure to calreticulin (CRT), secretion of ATP, and release of high-mobility group protein 1 (HMGB1). This process enhanced the effectiveness of NK cell-membrane immunotherapy. Notably, immunogenic PDT played a crucial role in augmenting the effects of NK cell-membrane immunotherapy.^69^ It significantly improved the infiltration of effector T cells, including CD4 + and CD8 + T cells, into tumors. Consequently, employing this integrated method effectively suppressed both the main tumors and distant tumors by inducing an abscopal effect (Figure 5).^68^

 The research conducted by Liu and colleagues addresses a notable challenge in the use of PDT for cancer treatment, namely the presence of hypoxia within the tumor microenvironment.^70^ To overcome this challenge, the researchers harness the potential of photosynthetic bacteria known as Synechococcus 7942 (Syne) to augment PDT. This is accomplished by establishing a biomimetic system, denoted as S/HSA/ICG, wherein HSA/ICG NPs (nanoparticles encapsulating indocyanine green (ICG) within human serum albumin (HSA)) are securely attached to the Syne surface. This innovative approach leverages both the photosynthetic capabilities of Syne and the therapeutic attributes of HSA/ICG. Upon administration into mice with tumor-bearing conditions, S/HSA/ICG accumulates within the tumors and, upon exposure to laser irradiation, continuously generates oxygen through photosynthesis, generating a substantial amount of oxygen and contributing to the release of ROS with photodynamic properties. Simultaneously, this oxygen production improved the hypoxic conditions within the tumor and reversed the immunosuppressive microenvironment. Most notably, the amelioration of tumor hypoxia further heightened the effectiveness of ICG in PDT and triggered ICD, resulting in an antitumor immune response, the application of HSA/ICG NP-coated Syne (S/HSA/ICG) brings about a synergistic inhibition of both local and metastatic tumors in a murine model of 4T1 mTNBC. This mechanism efficiently alleviates tumor hypoxia and amplifies the generation of ROS, ultimately leading to the total eradication of primary tumors.

## Conclusion

 PDT is getting attention as a complementary and alternative treatment option for various types of cancers and nanotechnology plays an important role in improving its efficiency. A liposomal drug delivery system for PS’s encapsulation has been widely used for PDT application. They have various advantages for improving PDT efficacy through enhancing bioavailability, increasing cell specificity, and preventing PS aggregation. Due to the presence of phospholipids’ hydrophilic head, liposomes have the ability to conjugate with antibodies which improve the efficacy of targeted PDT.

 Despite remarkable advancement in liposomal nanomedicine and PDT, still, challenges are there with respect to pharmacokinetic behavior, and tolerable properties of nano-drug carriers. Normally, 2D monolayer cell cultures are used to evaluate the efficacy of nano-drug carriers. These cultures lack the intrinsic tumor microenvironment and cell-to-cell interaction which affect the phenotypic discrepancies as compared to the real tumors. This suggests that numerous preclinical studies of liposomal products fail for clinical trials thus delaying the effective therapeutic strategies for cancer treatment. Keeping in view the above comments the 3D cell cultures could give promising bridging between preclinical and clinical trials of liposomal drug delivery systems for targeted PDT because they are similar to real tumors in various features. Subsequently, smart PS-loaded liposomal drug carriers having conjugation with targeted ligand and with combination strategies (chemo, radio, etc.) could contribute novel opportunities for clinical cancer treatment and enhance the therapeutic efficacy with minimum side effects.

## Competing Interests

 None declared.

## Ethical Approval

 Not applicable.
